# Automated bedside flow cytometer for mHLA-DR expression measurement: a comparison study with reference protocol

**DOI:** 10.1186/s40635-017-0156-z

**Published:** 2017-08-30

**Authors:** Mehdi Zouiouich, Morgane Gossez, Fabienne Venet, Thomas Rimmelé, Guillaume Monneret

**Affiliations:** 1 Hospices Civils de Lyon, Immunology Laboratory, E. Herriot Hospital, 69003 Lyon, France; 2 EA7426 “Pathophysiology of injury-induced immunosuppression” (University Claude Bernard Lyon 1 – Hospices Civils de Lyon – bioMérieux), 69008 Lyon, France; 3 Hospices Civils de Lyon, Anesthesiology and Intensive Care department, E. Herriot Hospital, 69003 Lyon, France; 4 Cellular Immunology Laboratory, Hôpital E. Herriot – Hospices Civils de Lyon, Pavillon E – 5 place d’Arsonval, 69437 Lyon Cedex 03, France

**Keywords:** Flow cytometry, mHLA-DR, Accellix, Automated flow cytometer, Immunosuppression, Sepsis, Septic shock

## Abstract

**Background:**

In various ICU conditions, measurement of diminished expression of human leukocyte antigen-DR on circulating monocytes (mHLA-DR) by flow cytometry appears to be a reliable marker of acquired immunosuppression. Low mHLA-DR is associated with an increased risk of nosocomial infections and mortality. Nevertheless, its use remains somewhat limited and has not been adopted in common medical practice. The main drawback of mHLA-DR measurement is likely related to the use of flow cytometry that is not accessible everywhere on a 24/7 basis. Recently, the Accellix system, a fully automated table top cytometer, was developed for use at bedside or emergency labs.

**Methods:**

The objective was to assess the performance of the Accellix (beta site evaluation including repeatability and method comparison with reference protocol) for the measurement of mHLA-DR expression.

**Results:**

Accellix repeatability at low and high expression levels of mHLA-DR was < 10% (i.e., within the range of acceptability for clinical flow cytometry). In comparison study including 139 blood samples (67 septic shock patients and 17 healthy volunteers), Pearson’s correlation parameters (*r* ^2^) ranged from 0.71 to 0.97 (*p* < 0.001). Intra-class correlation coefficient was 0.92.

**Conclusions:**

This fully automated table top cytometer appears to be a suitable tool for ICU patient monitoring and on-going clinical trials as there is no sample preparation and no need for specific skills in flow cytometry. Upon validation in a larger cohort study to reinforce reliability, Accellix could represent a major step to make flow cytometry accessible to clinicians by placing the instrument inside intensive care units or emergency laboratories.

**Electronic supplementary material:**

The online version of this article (10.1186/s40635-017-0156-z) contains supplementary material, which is available to authorized users.

## Background

Septic patients and critically ill patients (i.e., trauma, burns, and stroke) develop a complex immune response that may lead to a protracted immune-suppressive state. Magnitude and persistence of immunological alterations over time have been associated with poor clinical outcomes: development of nosocomial infections, viral reactivation, and increased mortality [1]. Consequently, novel stimulating approaches recently appeared as promising therapies [1]. As there is no clinical sign for immunosuppression, biomarkers are needed in order to both include patients and ideally to monitor response to treatment [2].

In various ICU conditions (e.g.*,* trauma, burns, sepsis), measurement of diminished expression of human leukocyte antigen-DR on circulating monocytes (mHLA-DR) by flow cytometry appears to be a reliable marker of acquired immunosuppression [3]. Low mHLA-DR monocytes exhibit altered functionality. Clinically, low mHLA-DR is associated with an increased risk of nosocomial infections and mortality [4]. Of note, mHLA-DR has already been used for patient stratification in a small GM-CSF trial that provided encouraging results [5]. Nevertheless, despite a large body of literature showing mHLA-DR as an excellent biomarker, its use has remained somewhat limited and has not been adopted in common medical practice. Its main drawback is likely related to the use of flow cytometry. Indeed, flow cytometry facilities are—if present at hospital—usually not always available (mainly business hours). The technology requires specialized technical skills in complex fields, which may be a hurdle for clinicians not familiar with cell analysis. Recently, the Accellix system, a fully automated table top cytometer requiring only introduction of the patient sample into a cartridge, was developed for use at the bedside or in emergency labs. Sample preparation and reading are performed in a dedicated disposable cartridge. Its first application was the measurement of neutrophil CD64 overexpression for diagnosis of bacterial infection [6]. The objective of the present study was to assess the analytical mHLA-DR measurement performance of Accellix cytometer. To this end, we mainly conducted a comparison study between Accellix values and those obtained with the standardized protocol [7].

## Methods

### Study population

The study group consisted of 67 septic shock patients (diagnostic criteria of the International Guidelines for Management of Severe Sepsis and Septic Shock admitted to the surgical ICU (E. Herriot Hospital – Lyon University hospitals). Seventeen healthy volunteers were included in the study after informed consent was given. Regarding septic patients, this work belongs to a global study on ICU-induced immune dysfunctions. It has been approved by our Institutional Review Board for ethics (“Comité de Protection des Personnes Sud-Est II” #IRB 11236) and registered at French Ministry of Research and Enseignement (#DC-2008-509). Each patient or his/her relative was orally informed about objectives and conduct of this clinical study and received a note summarizing this information. Patients were included only if the non-opposition to inclusion in the study had been expressed. This was recorded in each patient’s clinical file. For this non-interventional study assessing clinical data and biological samples, oral informed consent from the patient or a relative was considered sufficient. Written informed consent is not required by French law for studies carried out in current care.

### Cell preparation and flow cytometric analysis

Peripheral blood samples were obtained from routine monitoring workload collected in EDTA anticoagulant tubes: 139 samples from 67 septic patients, at different time points, and 17 healthy volunteers (flow chart in Additional file 1: Figure S1). Cells were stained according to the standardized protocol as fully described in 2005 [7, 8]. Briefly, 50 μL of whole blood was stained with 20 μL of QuantiBrite HLA-DR/Monocyte mixture (QuantiBrite anti-HLA-DR PE (clone L243)/Anti-monocytes (CD14) PerCP-Cy5.5 (clone MUP9), Becton Dickinson San Jose, CA, USA) at room temperature for 30 min in a dark chamber. Samples were then lysed with FACS Lysing solution (Becton Dickinson) for 15 min. After a washing step, cells were analyzed on Navios (Beckman Coulter). Monocytes were first gated out from other cells on the basis of CD14 expression, and mHLA-DR expression was then measured on their surface (mono-parametric histogram) as median of fluorescence intensity (MFI) related to the entire monocytes population (as recommended by the manufacturer). These results were then transformed to ABC (number of antibodies fixed per cell) using the response of calibrated PE-beads (BD QuantiBriteTM - PE Beads, Becton Dickinson). After staining onset, results are obtained in 60 min.

**Fig. 1:**
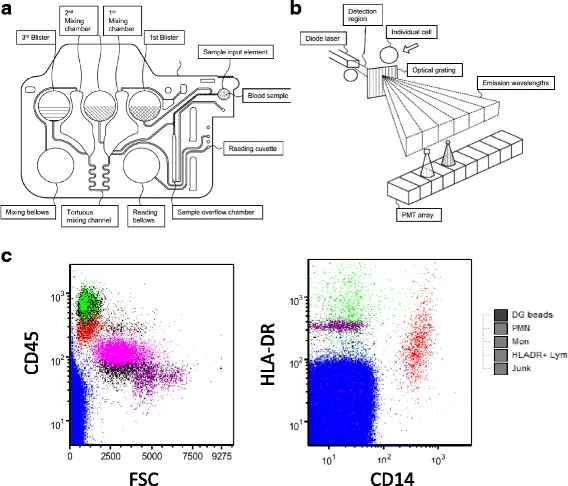
Key elements of the Accellix cartridge based HLA-DR flow cytometry assay. **a** Cartridge. The sample is added to the cartridge, which is then placed in the reader instrument. Reagents are added to the sample by crushing the 1st blister on the cartridge that releases the reagents and simultaneously moves the sample to a first mixing chamber. Additional mixing is performed by moving the composition of the sample and reagents from one mixing chamber to the other through a tortuous path by compressing and releasing a polymer bellows in order to alternately apply pressure and vacuum to the sample. After the incubation period, emission level reference beads are added by crushing the 3rd blister to release the beads into the 2nd mixing chamber. The resulting composition is again moved back and forth between the two mixing chambers in order to obtain a uniform particle suspension. At the end of this mixing process, the composition is in the first mixing chamber. The reading bellows, which had been previously compressed, is now released in order to apply vacuum to draw the specimen from the first mixing chamber through the reading cuvette. **b** Optical detection. Fluorescence emission detection is performed by dispersing the emission using an optical grating and collecting the dispersed emission using a PMT array. **c** Dot plot histograms. Results from the automatic classification algorithm are shown on the 2 plots. First, junk is excluded based on the absence of any fluorescent signal. Reference beads are identified because they do not express pan WBC CD45 marker. PMNs used as a negative control are identified based on the levels of CD45, CD14, and forward scatter. Finally, lymphocytes used as a positive control are separated from monocytes based on CD14 expression. Importantly, it is to note that these histograms are not accessible to Accellix users and are only provided here as an illustration of cell identification process

In parallel, when starting above staining, we deposited 40 μL of whole blood on an Accellix cartridge that was immediately placed in the reader instrument (Fig. 1). This constituted the sole technical step requiring human intervention. Accellix disposable mHLA-DR cartridge-based reagents are the following: anti-HLA-DR FITC, anti-CD45 PE, anti-CD14 PE-Cy5, and Dragon Green (DG) beads (similar to FITC). Briefly, the fully automated sample processing sequence taking place inside the cartridge and driven by the instrument begins with 10-min incubation with staining antibodies and lysis buffer (i.e., red blood cells lysis takes place during staining incubation). DG beads are added at the end of the staining/lysing process and are mixed just prior to reading the sample (Fig. 1). Additional details on reagents and algorithms are also provided in Additional file 1. Measurements were calculated as mHLA-DR index, i.e., the ratio between MFI of monocyte population and MFI of beads. These index values are adjusted to partially match the units of the BD QuantiBrite anti-HLA-DR PE results.

### Repeatability on Accellix

One patient and one healthy volunteer were included for the evaluation of repeatability. This was estimated as the coefficient of variation (% CV) of mHLA-DR measurements. In order to avoid the non-specific rise of mHLA-DR over time [9], blood samples were kept on ice and measurements were performed every 25 min (i.e., turnaround time on Accellix) during 3 h after sampling (i.e., *n* = 6 measurements [10]).

### Statistical analysis

CV, intra-class correlation coefficient (ICC), and Passing-Bablok correlation coefficient were calculated with R Studio software (Version 0.99.902). Sample size of this study was above the recommended threshold (i.e., > 32) for an agreement study [11].

## Results and discussion

**Fig. 2:**
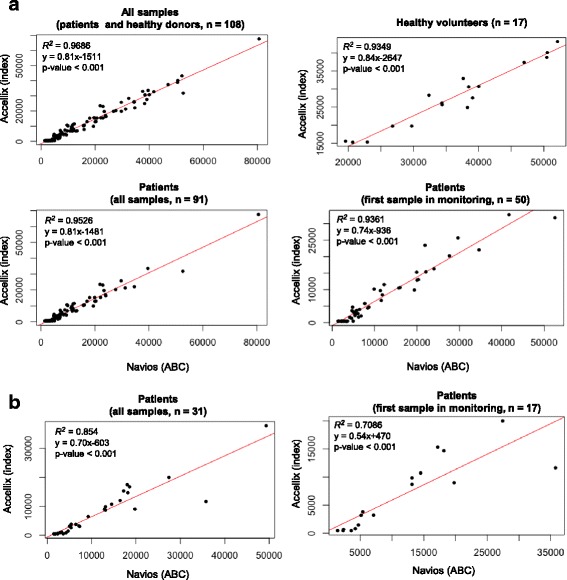
Comparison of mHLA-DR expression measurement between Accellix (mHLA-DR index) and Navios (ABC). **a** 108 blood samples (retrospectively analyzed with modified algorithm) as: upper left (all samples), upper right (only healthy volunteers), lower left (septic patients: all samples), lower right (septic patients: only first sample in the monitoring course). **b** 31 blood samples from 17 septic patients (prospectively analyzed) as: left (all samples), right (only first sample in the monitoring course)

Sixty-seven septic shock patients were included (53% male, mean age 72 years, mean SOFA 9, mean SAPS II 53, 28-day mortality 28%). Repeatability CV was within the range of acceptability (i.e., < 10%) regarding clinical flow protocols [10, 12] for both patient and healthy volunteer samples: 8.07% (mean of mHLA-DR index = 3766) and 1.32% (mean of mHLA-DR index = 42,644) obtained from 6 time points. As observed for other flow cytometry parameters, it is usual to observe higher CV with low values (e.g., CD4 counts; [12]). Overall, correlation between the two cytometers was satisfactory. First part of the study was conducted on 108 samples from 50 septic shock patients and 17 healthy volunteers. Correlation parameters were found to be *R* ^*2*^ = 0.77, *y* = 0.8192 × −552 (*p* < 0.001, linear model) and y = 0.8186×-1616 (Passing-Bablok regression). Nevertheless, 3 outliers were identified. In these cases, after carefully checking histograms from the usual flow cytometry protocol, Accellix values appeared to be in error. One likely reason was the presence of a significant neutrophil population with increased auto-fluorescence. To fix this issue, a new algorithm was developed and installed on Accellix. We could retrospectively reanalyze the first 108 samples. Correlation results are presented in Fig. 2a; there was no longer a discrepancy. For the whole cohort, the Pearson correlation parameters were found to be *R* ^*2*^ = 0.9686, y = 0.8099×-1511 (*p* < 0.001). ICC was also high at 0.92 indicating very good concordance between both measurements. When data were split to differentiate results obtained in healthy donors versus septic patients or when only one sample per patient was kept in the analysis (to decrease potential bias associated to serial sampling from the same patient), correlation parameters remained very good (Fig. 2a).

After installation of the modified algorithm, we prospectively included 32 additional blood samples from 17 septic patients. Correlation analysis of these samples yielded similar results to those obtained for the first 108 samples (*R* ^*2*^ = 0.85, y = 0.70×-603, *p* < 0.001; Fig. 2b). We did not identify any aberrant result.

Of note, correlation parameters (intercept − 1511 or − 538) suggest that Accellix results are lower than those from usual flow cytometry. This result is actually not perfectly accurate as limit of blank (LOB) for Accellix is about 450 (mHLA-DR index) corresponding to about 2400 ABC on Navios. Below this threshold, Accellix provided results as < LOB. In our analysis, when Accellix gave < LOB results, we plotted 450 to draw the curve. This may explain that for very low values of mHLA-DR, correlation is less good. Importantly, it should be kept in mind that this potential drawback would have no consequence in clinics. Indeed, to date, the cutoff used to stratify patients in immunostimulation trial is 8000 AB/C [5] which would approximately correspond to 5000 mHLA-DR index on Accellix (extrapolation from correlation curves), which is largely above Accellix LOB. Patient with < LOB Accellix values have to be considered very sick (at least, from an immunological perspective). With them, kinetics aspect would be what matters the most (i.e., is the patient recovering mHLA-DR expression over time?).

Finally, it is important to report that during repeatability study, we noticed a discrepancy for a single value (mHLA-DR index = 45,000 whereas all other values were about 3800). After checking, it was due to a fluidic issue that gave no error message. This obviously aberrant measurement was not taken into account in CV calculation. Since the flow cytometer did not provide any error message, this deserves further explorations from the manufacturer.

Overall, at this stage, except for this aberrant value out of 139 measurements, this first beta-site Accellix study regarding mHLA-DR assessment is very encouraging. Although we had no access to the algorithm and flow histograms, we noticed satisfactory results for a comparison study. In addition, despite known pre-analytical issues [9], repeatability results were found to be within acceptable range for flow cytometry. To date, mHLA-DR, although providing excellent information, has remained under-utilized due to the difficulty to use/have access to flow cytometry. In contrast, Accellix may provide a workable solution for this problem. The sole human intervention is to deposit about 40 μL of whole blood on the cartridge. As there is no flow histogram available on Accellix, there is no interpretation of the results based on usual gating strategy or fluorescence compensation. No specific technical skills in flow cytometry are thus required. Accellix provides a result as mHLA-DR index—nothing else. Thus, Accellix may be installed directly in ICUs or in 24/7 emergency labs where traditional flow cytometer are not present. Beyond routine monitoring, in the short term, it may facilitate managing multicentered clinical trials based on mHLA-DR patient stratification. To date, the main Accellix drawback lies in the fact that it can only analyze a single cartridge at a time and requires 25 min to do so. This implies a need to correctly schedule blood sampling and analysis onset when multiple samples are to be analyzed simultaneously as blood cannot be stored for too long before mHLA-DR staining. By nature, in a beta site evaluation, clinical decision-making thresholds are not investigated. Thus, a next important step would be to conduct a comparative study in a larger cohort of patients to define and assess those thresholds in predicting mortality and/or secondary infection occurrence and to evaluate how they interface with usual values from HLA-DR as ABC obtained with standard protocol.

## Conclusions

Overall, this fully automated table top cytometer may be a suitable tool for ICU patient monitoring and on-going clinical trials as there is no need for sample preparation and results are quickly obtained (under 30 min for each cartridges). As a first beta site evaluation, results are promising, and upon validation in a larger cohort study to reinforce reliability, Accellix could be a major step to make flow cytometry accessible to clinicians by placing the machine directly in intensive care units (ICU) or emergency laboratories. If first results of immunostimulation trials are encouraging, mHLA-DR on Accellix might become a very popular parameter.

## Additional file

Additional file 1: Figure S1. Flow chart and additional methods. (DOCX 67 kb)

## Abbreviations

AB/C: Antibody per cell
CD: Cluster of Differentiation
GM-CSF: Granulocyte Macrophage Colony-Stimulating Factor
HV: Healthy volunteer
ICU: Intensive care unit
LOB: Limit of blank
mHLA-DR: Human leukocyte antigen-DR on monocytes
