# Supplementary material for: Automated bedside flow cytometer for mHLA-DR expression measurement: a comparison study with reference protocol
Source: Intensive Care Med Exp. 2017 Aug 30;5:39. doi: 10.1186/s40635-017-0156-z (PMC5577346; doi:10.1186/s40635-017-0156-z)
Supplement: Additional file 1: Figure S1. — Flow chart and additional methods. (DOCX 67 kb) [file 40635_2017_156_MOESM1_ESM.docx]

**Intensive Care Medicine Experimental – Research Article**

**Additional file 1**

**Title:** Automated bedside flow cytometer for mHLA-DR expression measurement: a comparison study with reference protocol

**Authors:** Mehdi Zouiouich^1,2^, Morgane Gossez, PharmD^1,2^, Fabienne Venet PharmD, PhD^1,2^, Thomas Rimmelé, MD, PhD^2,3^, Guillaume Monneret, PharmD, PhD^1,2,4^

(1) Hospices Civils de Lyon, Immunology Laboratory, E. Herriot Hospital, Lyon, 69003, France

(2) EA7426 « Pathophysiology of injury-induced immunosuppression » (University Claude Bernard Lyon 1 – Hospices Civils de Lyon – bioMérieux), Lyon, 69008, France

(3) Hospices Civils de Lyon, Anesthesiology and Intensive Care department, E. Herriot Hospital, Lyon, 69003, France

(4) TRIGGERSEP network

**Email addresses:**

[mehdi.zouiouich@etu.univ-lyon1.fr](mailto:mehdi.zouiouich@etu.univ-lyon1.fr)

[morgane.gossez@chu-lyon.fr](mailto:morgane.gossez@chu-lyon.fr)

[fabienne.venet@chu-lyon.fr](mailto:fabienne.venet@chu-lyon.fr)

[thomas.rimmele@chu-lyon.fr](mailto:thomas.rimmele@chu-lyon.fr)

[guillaume.monneret@chu-lyon.fr](mailto:guillaume.monneret@chu-lyon.fr)

**Figure S1. Flow chart**

**
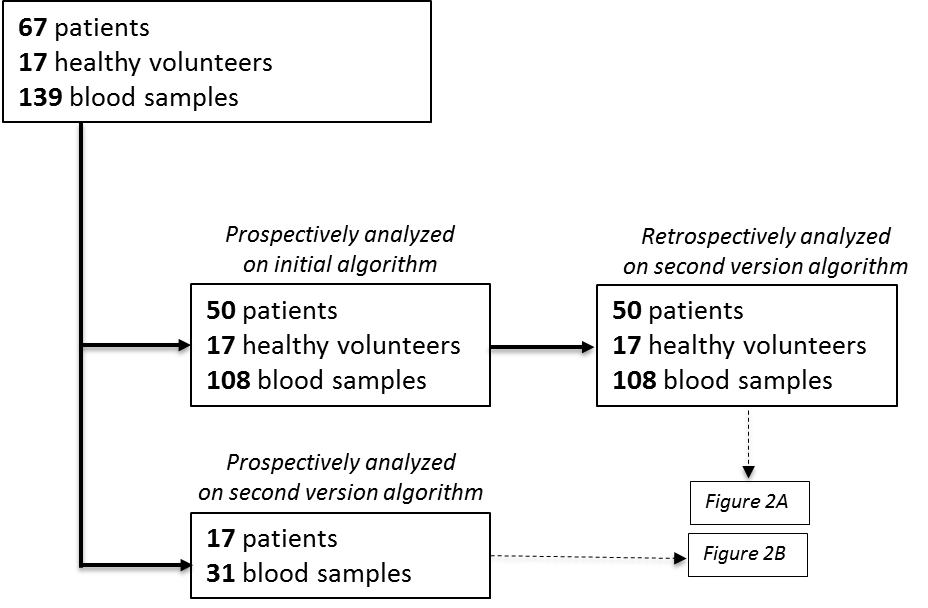
**

# Cartridge and Reagents

The following table summarizes the antibody reagents and fluorescent tags used in this assay. All of the antibodies along with a lysing reagent are stored in the 1^st^ blister in the cartridge. The 2^nd^ blister in the cartridge is not used, and the Dragon Green reference beads are stored in the 3^rd^ blister.

| **Reagent** | **Dye** | **Peak Channel** | **Marker Expressed by** |
| --- | --- | --- | --- |
| Anti-HLADR | FITC | CH2 | Monocytes and some Lymphocytes |
| Anti-CD45 | PE | CH4 | All WBCs (Lymphocytes, Monocytes, PMNs) |
| Anti-CD14 | PE-Cy5 | CH7 | Monocytes |
| DG beads  (15.4 uM) | Dragon Green  (similar to FITC) | CH2 | NA |

Briefly, the processing sequence is the following.

- Incubation with staining reagents is 10 minutes long.
- The reagents are stored in lysis buffer, so RBC lysis takes place during staining incubation.
- The DG beads are added at the end of the staining/ lysing process, and are mixed just prior to reading the sample.

# Optical Detection

Detection of the excited fluorescence emission is performed by dispersing the emission using an optical grating and collecting the dispersed emission using a PMT array. The 8 elements collect wavelengths from 500 through 750 nm.

# Algorithm

Individual events are determined from the amplitude of the forward scatter signal as each particle passes through the detection region. Each event is classified based on the amplitude of the signals present on the PMT elements. The essence of the automatic classification algorithm is summarized schematically as follows:

First, junk is excluded based on the absence of any fluorescent signal. DRG reference beads are identified because they do not express pan WBC CD45 marker. PMNs used as a negative control are identified based on the levels of CD45, CD14 and forward scatter. Finally, lymphocytes used as a positive control are separated from monocytes based on CD14 expression. Results of applying this algorithm to a sample are shown in Fig 1S C.
